# Comparison and Relative Utility of Inequality Measurements: As Applied to Scotland’s Child Dental Health

**DOI:** 10.1371/journal.pone.0058593

**Published:** 2013-03-08

**Authors:** Yvonne I. Blair, Alex D. McMahon, Lorna M. D. Macpherson

**Affiliations:** 1 Dental Public Health, Oral Health Directorate, Clutha House, Glasgow, Scotland, United Kingdom; 2 Community Oral Health Section, University of Glasgow Dental School, Glasgow, Scotland, United Kingdom; University of Toronto, Canada

## Abstract

This study compared and assessed the utility of tests of inequality on a series of very large population caries datasets. National cross-sectional caries datasets for Scotland’s 5-year-olds in 1993/94 (n = 5,078); 1995/96 (n = 6,240); 1997/98 (n = 6,584); 1999/00 (n = 6,781); 2002/03 (n = 9,747); 2003/04 (n = 10,956); 2005/06 (n = 10,945) and 2007/08 (n = 12,067) were obtained. Outcomes were based on the d_3_mft metric (i.e. the number of decayed, missing and filled teeth). An area-based deprivation category (DepCat) measured the subjects’ socioeconomic status (SES). Simple absolute and relative inequality, Odds Ratios and the Significant Caries Index (SIC) as advocated by the World Health Organization were calculated. The measures of complex inequality applied to data were: the Slope Index of Inequality (absolute) and a variety of relative inequality tests i.e. Gini coefficient; Relative Index of Inequality; concentration curve; Koolman & Doorslaer’s transformed Concentration Index; Receiver Operator Curve and Population Attributable Risk (PAR). Additional tests used were plots of SIC deciles (SIC^10^) and a Scottish Caries Inequality Metric (SCIM^10^). Over the period, mean d_3_mft improved from 3.1(95%CI 3.0–3.2) to 1.9(95%CI 1.8–1.9) and d_3_mft = 0% from 41.1(95%CI 39.8–42.3) to 58.3(95%CI 57.8–59.7). Absolute simple and complex inequality decreased. Relative simple and complex inequality remained comparatively stable. Our results support the use of the SII and RII to measure complex absolute and relative SES inequalities alongside additional tests of complex relative inequality such as PAR and Koolman and Doorslaer’s transformed CI. The latter two have clear interpretations which may influence policy makers. Specialised dental metrics (i.e. SIC, SIC^10^ and SCIM^10^) permit the exploration of other important inequalities not determined by SES, and could be applied to many other types of disease where ranking of morbidity is possible e.g. obesity. More generally, the approaches described may be applied to study patterns of health inequality affecting worldwide populations.

## Introduction

A major goal of the World Health Organisation is to eliminate the extensive, preventable and unjust health inequalities which persist within and between countries [1]. Efforts are being made to understand and address inequalities and regional and local policymakers and governments are increasingly demanding improvements in health and concurrent reductions in health inequality [2], [3],4]. Nevertheless, despite global ambitions to decrease population prevalence and simultaneously reduce inequalities, a consensus has yet to emerge as to how changes in inequality should be measured.

Inequality can be measured on an absolute or relative scale, and can be reported using straightforward or complex methods [5], [6]. The simple metrics compare only two groups on a SES index, usually the most disadvantaged to the most advantaged, or alternatively, the median group serves as the reference/comparator group. However, proponents of the complex methodologies would nowadays consider this to be an inadequate approach by which to measure or monitor health inequalities [2], [5], [6], [7]. This is because of the comparative dissonance between the simple methodology and contemporary concepts that health inequality is characterised by systematic relationships across gradients of relative advantage and disadvantage in the population [2]. Reduction of inequality calls for action on the social determinants of health across the whole population distribution and the principle of ‘*Proportional Universalism’* is fundamental to the design of effective strategies [1], [2]. Thus, comprehensive measurement and monitoring of inequalities makes it necessary that the whole distribution of the health outcome of interest is taken into account within the metrics adopted [2]. Otherwise, it is impossible to assess the total impact on inequality from changes to the determinants of health.

It is acknowledged that the choice of inequality measures can predicate outcomes [8] and that no single inequality test is ideal [9]. There is therefore consensus that a variety of inequality measures should be employed with any dataset [5], [6], [7], [10], [11] and judgement exercised about which concept of disparity to measure [11], [12]. Nevertheless, one should adopt the fewest inequality tests, which will enable the most complete and accurate interpretation.

There has been evidence of interest in dental health inequality for some time [13], [14], [15] and there have been a number of studies utilising individual tests of inequality in relation to child dental caries in developed and developing countries e.g. in Australia, Scotland, USA and Brazil [16], [17], [18], [19], [20], [21]. Estimation of the magnitude of inequality has included methods with and without a socio-economic status dimension and use of both simple and complex measures.

There are relatively few publications about measurement methodologies and few papers have explored the use of a variety of complex absolute and complex relative inequality metrics with caries data [12], [22], [23]. Previous studies have been limited by small numbers of subjects [22], simulated datasets [23] and data provided by non-calibrated dental examiners [24]. Thus, to date research in this area has provided only a limited understanding of oral health inequalities and the pathways that are required to address them whilst striving to improve population oral health. The WHO Report, 2003, [25] emphasised the need for development of methodologies to analyse outcomes of oral health promotion programmes and for associated capacity building.

The direct relationship between SES and health outcomes in early and later life are well documented [1], [3]. However, over time, accepted methodologies for quantifying poverty have evolved beyond concepts solely related to quantification of income and expenditure. The Overseas Development Institute [26] describes “nine fault-lines” in contemporary debate on this subject, which now includes concepts of e.g. social exclusion, vulnerability, resilience and relative deprivation. Notwithstanding this, low income is often used as a proxy for poverty [27] when individuals’ equivalised household incomes fall beneath a specific threshold, commonly 60% of the median [27]. With respect to child poverty in Scotland during the period of interest, Scottish Government estimates range from a prevalence of 14% (95%CI, 14%–17%) to 10% (95%CI, 8%–11%), respectively, living in absolute poverty, in 2003/04 and 2010/11 [27]. However, when housing costs are taken into account, the prevalence of absolute poverty in the respective years was 18% and 13%. The downward trend can be explained in part by a fall in Scotland’s equivalised median income between 2009/10 and 2010/11 [28] resulting in fewer children’s families falling beneath the lowered threshold.

This reflects the trend observed in the UK [29] which is further attributed to increased levels of lone-parents in work and increases in the level of welfare benefits paid to families with children, over the period [29]. However, having acknowledged the recent UK improvement, the Child Poverty Action Group predicts that the UK prevalence of child poverty will rise by 17% by 2020 [29].

In Scotland, national child oral health improvement programmes over the past decade [30], [31] have been associated with recent improvements in children’s dental health [32]. The proportion of Scotland’s 5-year olds with no obvious decay experience (%d_3_mft = 0) has increased from 45.1% in 2000 to 67.0% by 2012 and the mean d_3_mft morbidity score has decreased from 2.73 to 1.35 teeth affected in respective years [32].

It is therefore important to study impacts on associated inequality. However, the choice of metrics remains debatable [5], [6]. No comprehensive assessment of available inequality methodologies which may be appropriate for monitoring dental health outcomes, particularly during a period when dental health has been improving rapidly [32], has been published. The aim of this study was therefore to model selected tests of inequality with a very large cross-sectional caries dataset and to make recommendations for the future, in relation to appropriate measures for studying child dental health inequality at the national level.

## Materials and Methods

The study analysed eight datasets from the *circa* biennial repeated cross-sectional surveys involving randomised samples of elementary schools and children aged 5-years-old, resident in respective NHS Board areas (currently, n = 14). This process produces substantial representative population sample fractions at the Scotland level, range 9.5%–24.9%, mean 15.4% per annum, over the period 1993/94–2007/08.

These population surveillance surveys, conducted by trained and calibrated dental examiners used the standardised diagnostic criteria of The British Association for the Study of Community Dentistry (BASCD) to measure dental caries at the level of visible penetration into the dentine layer of teeth, or beyond [33]. The d_3_mft index is the standard metric reporting caries epidemiology [25], [34] with lower case denoting deciduous teeth. The d_3_, m and f components denote the number of teeth that are decayed, missing (i.e. extracted due to decay) or filled, respectively.

These data are collected routinely by the National Health Service as part of a statutory dental inspection programme and comply with the legislation in Scotland with respect to informed, negative consent. Children’s home postcodes permitted calculation of SES deprivation (DepCat, 2004) categories [35] and linkage to subject’s caries data. DepCat is derived from categories created from the following variables collected at the national decennial census at postcode sector level i.e. proportions of: residents living in overcrowded households; unemployed males; persons in households headed by someone of low social class and persons who do not own a car. DepCat correlates consistently with morbidity and mortality data and is long established in Scotland [36], [37] as a composite area-based indicator of socioeconomic status (SES).

Logistic regression models used d_3_mft, age, gender, DepCat score and survey year as independent explanatory variables for d_3_mft>0. The following statistical methods were utilised: adjusted odds ratios (OR, 95%CI) for d_3_mft>0; Wilcoxon tests for d_3_mft scores and linear models. Simple measures of absolute and relative inequality in d_3_mft outcomes were calculated by comparing the values in the extreme DepCat groups. The odds ratio results for prevalence of d_3_mft>0 were used as an additional measure of simple relative SES inequality.

The Significant Caries Index (SIC) score [38] was calculated by ranking d_3_mft scores of all individuals, irrespective of their SES using a 33% cutpoint. The SIC score is the mean d_3_mft of the highest third of the distribution. We additionally used a modified SIC decile (SIC^10^) score using the highest tenth of the distribution of d_3_mft, after the methodology of Morgan *et al*. [39]. The odds-ratio for d_3_mft>0 were calculated using the most affluent DepCat group as the referent category.

The estimation of complex inequality in dental health focused on previously published tests i.e. the Gini coefficient [5] (estimated from a Lorenz Curve describing cumulative distribution of d_3_mft score), concentration curves [6] (CCs, examine the cumulative distribution of a health event (d_3_mft>0) with population ranked by SES group), the Concentration Index [6] (CI, computed from the area under the CC), Koolman & Doorslaer’s transformed CI [40] (the x75 multiplication of the CI produces a metric quantifying the % of health which would need to transfer from the relatively advantaged to the disadvantaged, to produce equity), the Slope Index of Inequality [6] (SII, based on regression of the mid-point value of d_3_mft score for each SES group across the cumulative distribution), Relative Index of Inequality [6] (RII, a relative version of the SII) and Population Attributable Risk [6], [10] (PAR, describes the proportion of d_3_mft>0 which could be prevented across all SES groups, if the prevalence in the most SES advantaged group could be generalised). Moreover, the Receiver Operating Characteristic (ROC, plots the sensitivity to 1 minus specificity for exposure to d_3_mft, with ranked SES scores, to give the predictive potential of SES for d_3_mft) analysis [41] was included. A further novel inequality metric has used the full distribution of SIC^10^ score for each population decile and has calculated the area under the curve of the SIC^10^ distribution for use as a single value Scottish Caries Inequality Metric (SCIM^10^). The SCIM^10^ value measures caries inequality between individuals across the whole population age group, without reference to SES. All analyses were carried out using SAS version 9.1 (SAS, Cary, NC). Locally written programming for each inequality test was validated [42].

## Results

**Figure 1:**
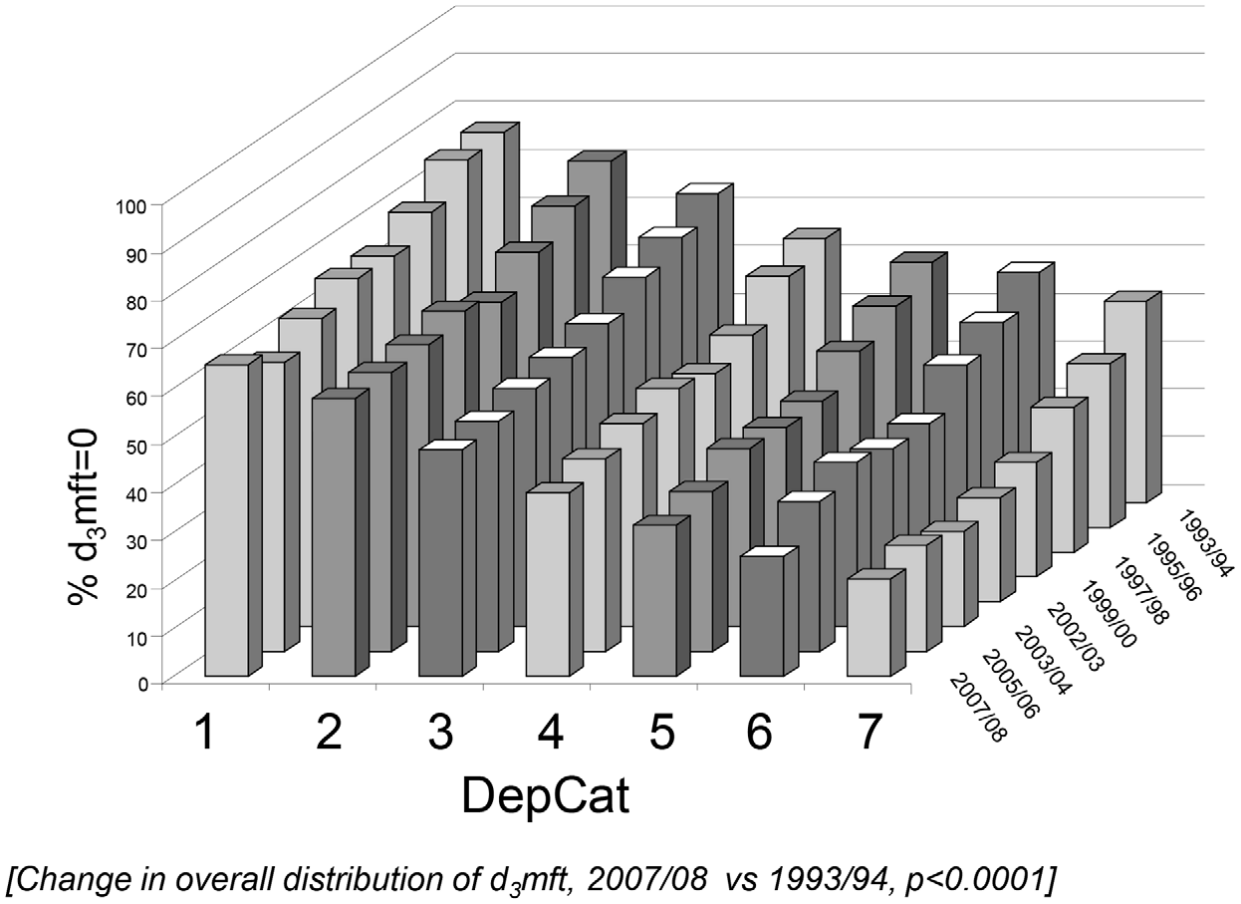
Prevalence of decayed, missing and filled teeth (%d_3_mft = 0) by SES for Scotland’s five-year-olds, 1993/94–2007/08. Prevalence of decayed, missing and filled teeth (%d_3_mft = 0) by SES for Scotland’s five-year-olds, 1993/94–2007/08. *(Change in overall distribution of d_3_mft, 2007/08 vs 1993/94, p<0.0001).*

The number of subjects included was n = 68,398. Figure 1 illustrates the trends in the prevalence of 5-year-olds with no decayed, missing or filled teeth (i.e. %d_3_mft = 0) by SES status, and Table 1 provides mean d_3_mft scores by SES and year. Age (p<0.0001), sex (p = 0.0007) and DepCat (p<0.0001) had the potential to confound effects. The adjusted odds-ratios (and 95% confidence intervals) demonstrated significant improvements over time in each SES domain for the prevalence of d_3_mft>0 (p<0.0001) and mean d_3_mft scores (Wilcoxon tests, p<0.001).

**Table 1:** Mean decayed, extracted and filled teeth scores (d_3_mft) for 5-Year-Old Children in Scotland, 1993/94–2007/08.

	Mean d_3_mft							
	Deprivation Category							
Year	1	2	3	4	5	6	7	Total (95% CI)
**1993/94**	1.3	1.8	2.7	3.2	3.7	4.1	5.0	3.1 (3.0–3.2)
**1995/96**	1.5	1.8	2.4	3.0	3.5	3.8	4.7	2.9 (2.8–3.0)
**1997/98**	1.3	1.5	2.2	2.7	3.1	3.5	5.0	2.7 (2.6–2.8)
**1999/00**	1.2	1.6	2.1	2.6	3.0	3.6	4.8	2.6 (2.5–2.7)
**2002/03**	1.2	1.8	2.2	3.0	3.3	3.8	4.5	2.8 (2.7–2.8)
**2003/04**	1.1	1.5	1.8	2.7	3.0	3.3	4.1	2.5 (2.4–2.5)
**2005/06**	0.8	1.3	1.7	2.2	2.7	2.9	3.8	2.2 (2.1–2.2)
**2007/08**	0.7	1.1	1.4	1.9	2.4	2.6	3.2	1.9 (1.8–1.9)

(Deprivation Category 1 = least deprived).

### Simple SES Inequality

Simple absolute SES inequality decreased with respect to the prevalence of decayed, missing and filled teeth (% d_3_mft>0) and mean d_3_mft scores. However, the associated simple relative SES inequality increased (Table 2). The odds-ratio for the experience of decayed, missing and filled teeth (d_3_mft>0) decreased from 7.5 (5.2–10.7) in 1993/94 to 4.9 (3.9–6.7) in 2007/08 when comparing the most deprived DepCat group to the most affluent (Table 2).

**Table 2:** Results from the application of a variety of inequality metrics to the decayed, missing and filled teeth scores (d_3_mft) from respective cross-sectional surveys of Scotland’s 5-year-olds, 1993/4–2007/8.

Simple SES Inequality in d_3_mft						Non-SES Based		Complex Inequality		
	mean		% >0							
year	Ab	Rel	Abs	Rel	OR (95%CI)	SIC	SIC^10^	SCIM^10^	K&D	Gini
**1993/94**	3.7	3.85	44.6	2.28	7.5 (5.2–10.7)	7.92	11.87	26.22	8.4	0.63
**1995/96**	3.2	3.13	38.1	1.96	5.4 (4.0–7.4)	7.5	11.14	24.70	7.7	0.63
**1997/98**	3.7	3.85	44.5	2.25	7.6 (5.7–10.0)	7.09	10.91	22.46	8.3	0.65
**1999/00**	3.6	4.0	45.7	2.4	7.7 (5.6–10.4)	6.64	10.43	20.30	8.9	0.67
**2002/03**	3.3	3.75	43.1	2.3	6.4 (4.8–8.5)	7.32	10.79	23.78	8.0	0.64
**2003/04**	3.0	3.71	40.5	2.39	5.6 (4.4–7.0)	6.77	10.33	20.56	9.1	0.68
**2005/06**	3.0	4.75	42.7	2.83	6.6 (5.2–8.2)	5.98	9.67	16.78	10.0	0.72
**2007/08**	2.5	4.57	35.3	2.54	4.9 (3.9–6.7)	5.43	9.27	14.49	9.8	0.74
**Trend**	p = 0.014	p = 0.055	p = 0.268	p = 0.035		p = 0.004	p<0.001	p = 0.004	p = 0.026	p = 0.005

**Abbreviations:**

Abs = Absolute inequality.

Rel = Relative inequality.

OR = Odds Ratio for d_3_mft>0 comparing most deprived (DepCat 7) with least deprived (DepCat 1).

SIC = Significant Caries Index.

SIC^10^ = Significant Caries Index of poorest decile.

SCIM^10^ = Scottish Caries Inequality Metric.

K&D = Koolman & Doorslaer’s Transformed Concentration Index.

### Complex SES Inequality

**Figure 2:**
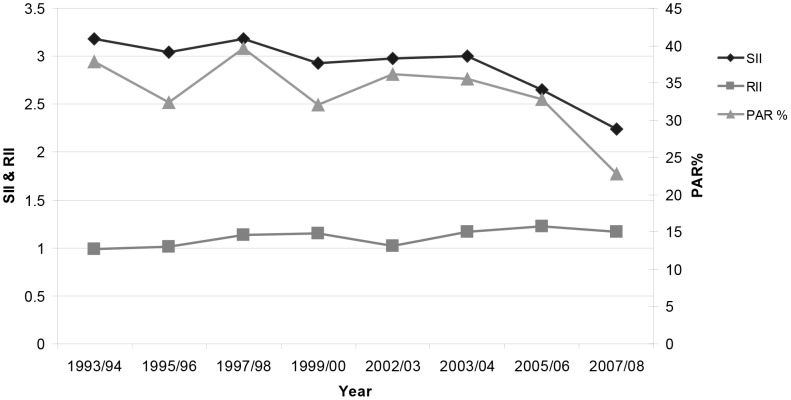
Slope Index of Inequality, Relative Index of Inequality and Population Attributable Risk for caries experience. Slope Index of Inequality (SII) and Relative Index of Inequality (RII) for d_3_mft score and Population Attributable Risk (PAR) for caries experience (%d_3_mft>0) in Scotland’s 5-year-olds, 1993/94–2007/08.

**Figure 3:**
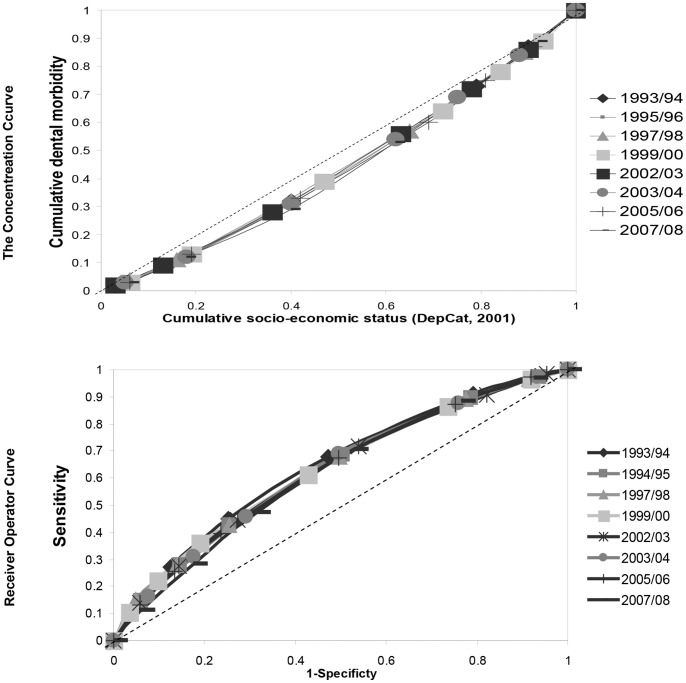
Concentration curves and Receiver Operator Curve plots for decay experience by SES. Concentration curves (CC) for d_3_mft scores (A) and Receiver Operator Curve (ROC) Plots for five-year-olds’ d_3_mft>0 (B) by SES (DepCat 2001) over the period 1993/94–2007/08.

The trend in SII (Figure 2), indicates that the complex absolute SES inequality improved from 1993/94–2007/08 (p = 0.012). The RII value increased marginally (Figure 2) over the interval (p = 0.045), with the complex relative SES inequality in caries experience outcomes remaining comparatively stable against the background of marked dental health improvement (Figure 1 & Table 1). Furthermore, the ROC plots and concentration curves (Figure 3) together with the Koolman and Doorslaer’s Transformed CI (Table 2) altered little over the interval. The results for the PAR (Figure 2) suggest that overcoming relative SES deprivation would itself have removed 37.9% of the population with experience of caries, extracted or filled teeth (d_3_mft>0) in 1993/94 and 22.8% in 2007/08 i.e. latterly, the prevalence of d_3_mft>0 has been modified to some extent and complex SES related inequality in caries prevalence has decreased (by this measure).

### Non-SES-based Tests of Inequality

**Figure 4:**
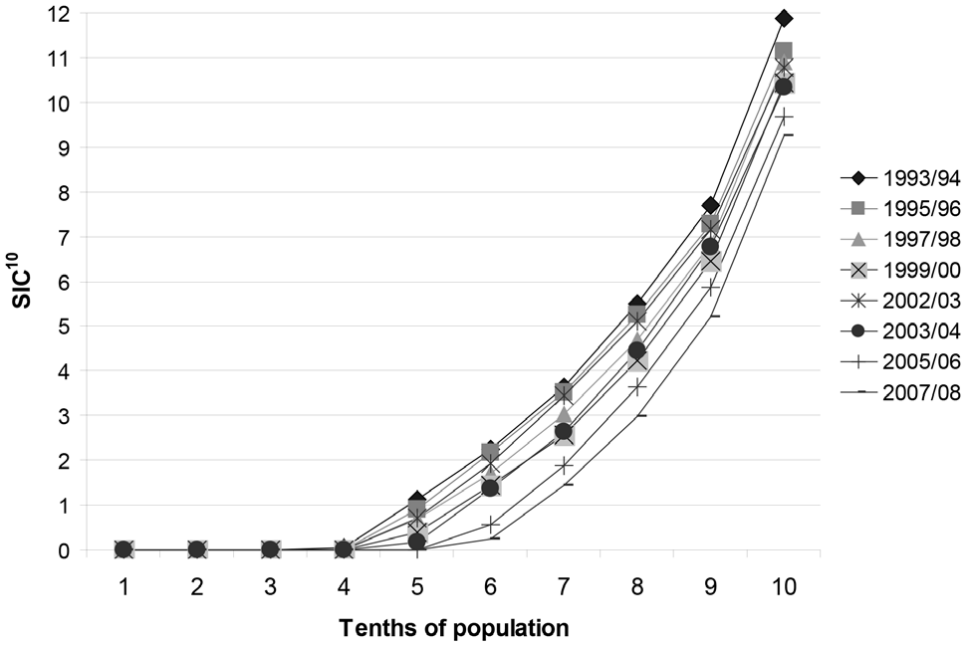
Significant Caries Index deciles (SIC^10^) for Scotland’s 5-year-olds, 1993/94–2007/08.

The Gini coefficients based on the d_3_mft scores (Table 2) indicate that, without reference to SES, there is an increase in the relative whole population inequality in dental health associated with decreasing prevalence of d_3_mft. However, the SIC and SIC^10^ scores (Table 2) along with the full SIC^10^ distribution (Figure 4) would seem to contradict this. The SIC^10^ distribution demonstrates that in each affected population decile (without reference to SES) there has been year-on-year reduction in both overall prevalence and burden of d_3_mft in affected individuals. The area under the SIC^10^ distribution curve i.e. the Scottish Caries Inequality Metric (SCIM^10^ score) has decreased significantly from 26.2 to 14.5 from 1993/94 to 2007/08, respectively.

## Discussion

This study is based on a very large dataset (n = 68,398) collected by trained and calibrated dental teams using standardised examination conditions and criteria over eight cross-sectional times points. Significant decreases in caries prevalence and morbidity were observed across the whole SES spectrum in Scotland in temporal association with implementation of the national (dental) health improvement policy-framework which commenced following publication of *Scotland’s Health a challenge to Us All* in 1995 [43], [44], [45], [46], [47]. The influential Marmot Report [2] suggests that it is necessary to monitor inequality across the SES spectrum to demonstrate whether the twin aims of improved overall population (dental) health and inequality outcomes have been achieved.

While inspection of the caries epidemiological trends by DepCat and the use of the conventional tests i.e. odds-ratios and Wilcoxon tests, permit us to be confident that caries prevalence and morbidity have decreased across the SES spectrum, these data alone do not adequately inform readers on the ways that caries inequality across the whole population may have changed. Formal tests of complex inequality are required for this [10]. Although the improvement in population health is a justifiable goal, there are nonetheless examples of well intentioned interventions which increased SES inequality in children’s dental health outcomes [48]. Modeling of selected tests of complex inequality with national caries datasets has permitted a thorough investigation of several different dimensions of inequality associated with changed dental health outcomes.

### Simple Inequality

The simple absolute and relative inequality results compared only two SES groups respectively and make no use of the data from across the SES spectrum. The odds ratio (OR) with 95% confidence interval provides useful statistical perspective on the changing magnitude of difference in prevalence between the most SES challenged group and the most advantaged counterpart at each cross-sectional point in this study. Nevertheless, although there is precedent for use of OR to estimate cross-section inequality in caries outcomes [21], this metric is not a conventional test of complex inequality. Thus, the reporting of simple inequality should always be accompanied by measurements of both complex absolute inequality and complex relative inequality to make use of all available data and take account of respective population sizes in the SES domains.

### Complex Inequality

Because of their ability to reflect the entire SES distribution and weight for population share in the respective SES groups, the SII and RII are recommended as good all round indicators of complex absolute and complex relative inequality, respectively [2], [7], [49]. The SII may be interpreted as the absolute difference overall in d_3_mft score when moving across the SES spectrum from the highest to the lowest SES group, which nonetheless is indicative of the total experience of individuals in the whole population. Moreover, SII is considered to be a consistent indicator with local populations [50]. In this study, the downward trend in SII has been most notable latterly. Alternatively, the RII may be interpreted as the SII relative to the overall mean d_3_mft of the weighted SES group values. RII is considered useful for making comparisons between different geographic places or cohorts [51]. Furthermore, there is a view that RII is less influenced by extremes of the outcome distribution [51].The literature on health inequality suggests that it is much more difficult to achieve improvements in relative inequality than improvements in absolute inequality, especially when the prevalence/morbidity in the denominator group/domain is decreasing. It is thus reassuring that the complex absolute inequality improvements noted herein have not been at the expense of large deteriorations in complex relative SES inequality. These Scottish findings are thus at odds with deciduous caries inequality trends observed in Australia [12]. However, it will be very difficult to decrease relative SES inequality with such a low prevalence in the comparatively affluent groups.

The CC and ROC plots capture ‘complex relative SES inequality’ across DepCat domains and provide useful visual imagery for quantifying relative SES inequality. Results for these tests suggest that in spite of national directed population dental health improvement interventions temporally associated with improved population d_3_mft outcomes [30], [31], [32], the causes of national relative SES inequality in caries outcomes continued to operate to a similar extent. The stability of CC and ROC plots show that the predictive potential of home DepCat score for prevalence of decayed, extracted and filled teeth (d_3_mft>0) and d_3_mft scores has remained remarkably constant.

The causal processes for dental caries morbidity are comparatively well understood from a scientific perspective, however, it must be borne in mind that the causes of relative caries inequality lie elsewhere, and are rooted in early life-course within the socioeconomic and psychosocial domains. Intervention measures which control caries prevalence and caries morbidity at population level may, or may not, impact on relative SES inequality. The foregoing CC and ROC results suggest that whatever in Scotland were the social determinants of 5-year-olds’ caries relative SES inequality in 1993/94, continued to exert effects. The depictions of the ROC and the CC should aid understanding and incorporation of at least one into reports would be beneficial. Koolman & Doorslaer’s transformed CI values may be interpreted as the percentage of health which the comparatively affluent would have to forgo to achieve equity by this measure. Reporting of the Transformed CI could be helpful in assessing the effectiveness on relative SES inequality of ‘proportionate universalism’ i.e. more resource-intensive interventions for those with greatest need, envisioned by Marmot *et al*. [2].

The PAR trend which measures complex relative SES inequality shows co-linearity with the SII. The former is based on prevalence scores, the latter on caries morbidity scores. Nevertheless, both take account of population size in the SES domains. However, derived from the field of epidemiology and interpreted differently, the PAR gives an indication of the proportion of those with experience of dental caries, extractions or fillings (d_3_mft>0) which could be eliminated if SES deprivation was eradicated (relative to the DepCat 1, reference group). The PAR is able to give quantity to the extent to which d_3_mft>0 prevalence within a population is attributable to SES deprivation. Although the mechanism of association remains unclear, the decreasing PAR values observed herein permit some optimism, as they suggest that the SES determinants of relative dental health inequality are not intractable in this instance.

### Non-SES-based Tests of Inequality

The Gini coefficient predominates in the generic inequality literature to measure economic inequality, for which it is extremely suitable. However, as the occurrence of d_3_mft becomes less dispersed and prevalent in a population (as is socially desirable and the aim of policy makers), Gini coefficient values indicate increasing inequality [24]. Thus, interpretation of Gini coefficient values with d_3_mft data against a background of improving population dental health with decreasing prevalence is far from intuitive (Table 1). The results for SIC and SIC^10^ scores give information with respect to d_3_mft scores among individuals within the worst third and tenth of a population’s caries distribution and could identify ‘at-risk’ groups, irrespective of SES. The modified SIC^10^ distribution and SCIM^10^ are not conventional tests of inequality in caries outcomes. Nevertheless, both provide intuitive information on inequality in distribution of d_3_mft>0. In common with the Gini coefficient, the SIC^10^ distribution has potential to inform on the total inequality between individuals. Examination of the whole SIC^10^ distribution permits review of inequality in the dispersion of decayed, extracted and filled teeth (d_3_mft) counts across all individuals in the population. This could be important when factors other than SES are relevant e.g. geography, ethnicity and language and it is not possible to rank variables on a scale. The strength of the direct systematic relationship between SES and caries outcomes in Scotland requires that SES is taken into account. However, in other countries ethnicity/race may be important considerations. Moreover, there is a view that to only consider SES inequality imposes a value judgement on data/outcomes and there are proponents of a ‘whole population’ view of inequality [52]. To date, the metric of choice with which to review total inequality would have likely been the Gini-coefficient [17], [24]. However, this test has the aforementioned shortcomings which render it intrinsically unsuitable for use with caries epidemiological data, as it has serious potential to mislead [24] and confuse government/policy makers [42]. Alternatively, the SIC^10^ distribution permits ‘at a glance’ assessment of the population prevalence of d_3_mft>0 and the mean d_3_mft count in population deciles. Moreover, the calculation of the area under the SIC^10^ curve provides the SCIM^10^ score which is a reliable single value metric with which to quantify whole population dispersion of decayed, extracted and filled teeth (d_3_mft). This novel test is useful for capturing simultaneously any changes in quantity and dispersion of caries morbidity in the affected deciles, over time. Furthermore, the SCIM^10^ can be interpreted intuitively.

### Conclusions

We have provided an insight into how inequalities in oral health might be considered. Our study has the advantage of being based on a series of very large population inspections in an area of historically poor oral health. When presenting caries inequality results, full understanding always necessitates showing the overall epidemiological data together with the simple and complex inequality results. Our results support the use of the SII and RII to measure complex absolute and relative inequalities alongside additional tests of complex relative inequality such as PAR and Koolman and Doorslaer’s transformed CI. The latter two tests have a clear interpretation and may influence policy makers. Moreover, the specialised dental metrics (i.e. SIC, SIC^10^ and SCIM^10^) permit exploration of inequalities that are not determined by SES, and of course could be applied to many other types of disease where ranking of morbidity is possible, such as hypertension, obesity and lung function. The approach adopted herein can be generalised to the study of patterns of health inequality within and between worldwide populations.
